# (*E*)-1-(2,5-Dimethyl-3-thien­yl)-3-(2,4,5-trimeth­oxy­phen­yl)prop-2-en-1-one

**DOI:** 10.1107/S1600536810028709

**Published:** 2010-07-24

**Authors:** Abdullah M. Asiri, Salman A. Khan, M. Nawaz Tahir

**Affiliations:** a The Center of Excellence for Advanced Materials Research, King Abdul Aziz University, Jeddah 21589, PO Box 80203, Saudi Arabia; b Department of Chemistry, Faculty of Science, King Abdul Aziz University, Jeddah 21589, PO Box 80203, Saudi Arabia; c University of Sargodha, Department of Physics, Sargodha, Pakistan

## Abstract

In the title compound, C_18_H_20_O_4_S, the thio­phene and benzene rings are oriented at a dihedral angle of 10.83 (11)°. The central chain makes dihedral angles of 1.86 (13) and 9.25 (12)° with the benzene and thio­phene rings, respectively. In the crystal, mol­ecules are linked through weak inter­molecular C—H⋯O inter­actions. π–π inter­actions are also observed between the benzene rings with a centroid–centroid distance of 3.6832 (12) Å. The slippage between the benzene rings is 0.956 Å.

## Related literature

For the biological activity of 1,3-diphenyl-2-propene-1-ones, see: Gökhan-Kelekçi *et al.* (2007 ▶); Ducki *et al.* (2009 ▶); dos Santos *et al.* (2008 ▶); Hussain *et al.* (2009 ▶); Dandia *et al.* (2006 ▶); Valla *et al.* (2006 ▶); Ye *et al.* (2004 ▶). For related structures, see: Asiri *et al.* (2009 ▶): Hussain *et al.* (2010 ▶): Fun *et al.* (2010 ▶).
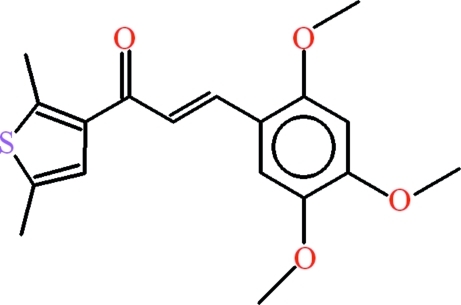


## Experimental

### 

#### Crystal data

C_18_H_20_O_4_S
*M* *_r_* = 332.40
Tetragonal, 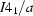

*a* = 19.5263 (5) Å
*c* = 17.9952 (4) Å
*V* = 6861.2 (3) Å^3^
*Z* = 16
Mo *K*α radiation
μ = 0.21 mm^−1^
*T* = 296 K
0.26 × 0.18 × 0.16 mm

#### Data collection

Bruker KAPPA APEXII CCD diffractometer
Absorption correction: multi-scan (*SADABS*; Bruker, 2005 ▶) *T*_min_ = 0.966, *T*_max_ = 0.975
25995 measured reflections
3106 independent reflections
2225 reflections with *I* > 2σ(*I*)
*R*_int_ = 0.038

#### Refinement

*R*[*F* ^2^ > 2σ(*F* ^2^)] = 0.040
*wR*(*F* ^2^) = 0.113
*S* = 1.05
3106 reflections
213 parameters
H-atom parameters constrained
Δρ_max_ = 0.19 e Å^−3^
Δρ_min_ = −0.15 e Å^−3^

### 

Data collection: *APEX2* (Bruker, 2009 ▶); cell refinement: *SAINT* (Bruker, 2009 ▶); data reduction: *SAINT*; program(s) used to solve structure: *SHELXS97* (Sheldrick, 2008 ▶); program(s) used to refine structure: *SHELXL97* (Sheldrick, 2008 ▶); molecular graphics: *ORTEP-3 for Windows* (Farrugia, 1997 ▶) and *PLATON* (Spek, 2009 ▶); software used to prepare material for publication: *WinGX* (Farrugia, 1999 ▶) and *PLATON*.

## Supplementary Material

Crystal structure: contains datablocks global, I. DOI: 10.1107/S1600536810028709/si2278sup1.cif

Structure factors: contains datablocks I. DOI: 10.1107/S1600536810028709/si2278Isup2.hkl

Additional supplementary materials:  crystallographic information; 3D view; checkCIF report

## Figures and Tables

**Table 1:** Hydrogen-bond geometry (Å, °)

*D*—H⋯*A*	*D*—H	H⋯*A*	*D*⋯*A*	*D*—H⋯*A*
C9—H9*C*⋯O3^i^	0.96	2.55	3.209 (3)	126
C14—H14⋯O4^ii^	0.93	2.57	3.483 (3)	168

Symmetry codes: (i) 
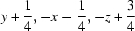
; (ii) 
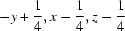
.

**Fig. 1.**
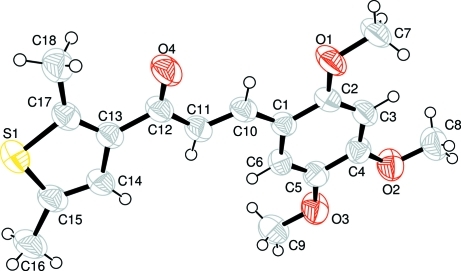
View of the title compound with the atom numbering scheme. The thermal ellipsoids are drawn at the 50% probability level. H-atoms are shown as small spheres of arbitrary radii.

**Fig. 2.**
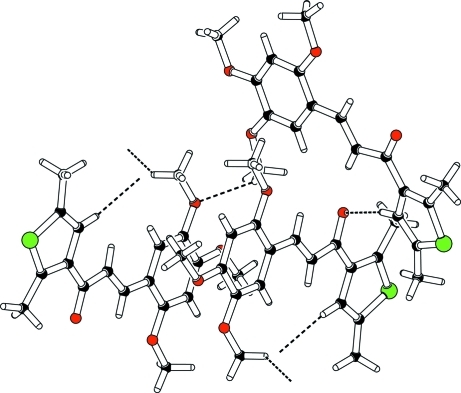
The partial packing (PLATON; Spek, 2009) which shows that molecules are interlinked through H-bondings.

## supplementary crystallographic information

### Comment

1,3-Diphenyl-2-propene-1-one, are considered to be precursors of flavonoids when found as naturally occurring compounds, but it could be considered that their true importance is extended in two branches: The biological activity associated with them, including anti-inflammatory (Gökhan-Kelekçi *et al.*, 2007), antimitotic (Ducki, *et al.*, 2009), anti-leishmanial (dos Santos, *et al.*, 2008), anti-invasive (Hussain, *et al.*, 2009), anti-fungal (Dandia *et al.*, 2006) antimalarial (Valla *et al.*, 2006) and anti-tumor (Ye *et al.*, 2004) properties; as well as their recognized synthetic utility in the preparation of pharmacologically interesting heterocyclic systems. On the bases of these aspects in this paper we are reporting the synthesis and crystal structure of the title compound (I), (Fig. 1).

The crystal structures of (II) *i.e.*, (2E,2^'^*E*)-1,1^'^-bis(2,5-dimethyl-3-thienyl)-3,3^'^-(*p*-phenylene) diprop-2-en-1-one (Asiri *et al.*, 2009) has been published which contain the 2,5-dimethylthiophen-3-yl moiety. Similarly, the crystal structures of (III) 2,3-dimethyl-*N*-[(*E*)-2,4,5-trimethoxybenzylidene]aniline (Hussain *et al.*, 2010) and (IV) 4-[(*E*)-(2,4,5-trimethoxybenzylidene)amino]-1,5-dimethyl-2- phenyl-1*H*-pyrazol-3(2*H*)-one (Fun *et al.*, 2010) have been published which contain the 2,4,5-trimethoxyphenyl moiety.

In (I), the group A (C1—C6/O1/O2/O3) of 2,4,5-trimethoxyphenyl moiety, the central chain B (C10—C12/O4) and 2,5-dimethylthiophen-3-yl C (C13—C18/S1) are planar with r. m. s. deviation of 0.0033, 0.0160 and 0.0031 Å, respectively. The dihedral angle between A/B, A/C and B/C is 1.80 (10), 10.65 (9) and 9.23 (12)°, respectively. Overall 2,4,5-trimethoxyphenyl group has a maximum deviation 0.0338 Å and in it the C9 deviates at maximum 0.0834 (20) Å. The molecules are interlinked through H-bondings of C—H···O type (Table 1, Fig. 2). There exist π—π interaction between the centroids of phenyl rings at a distance of 3.6832 (12) Å [symmetry: - *x*, - *y*, 1 - *z*].

### Experimental

A solution of 3-acetyl-2,5-dimethythiophene (0.38 g, 0.0025 mol) and 2,4,5-trimethoxy benzaldehyde (0.49 g, 0.0025 mol) in ethanolic solution of NaOH (3.0 g in 10 ml of methanol) was stirred for 16 h at room temperature. The solution was poured into ice cold water of pH = 2 (pH adjusted by HCl). The solid was separated and dissolved in CH_2_Cl_2_, washed with saturated solution of NaHCO_3_ and evaporated to dryness. The residual was recrystallized from methanol/chloroform to affoard yellow prisms.

Yield: 72%; m. p. 380–381 K.

IR (KBr) v_max_ cm^-1^: 3016 (Ar—H), 2924 (C—H), 1642 (C═O), 1572(C═ C). 1H NMR (DMSO-*d*_6_) (δ/p.p.m.): 8.01 (s, 1H, CH_aromatic_), 7.98 (s, CH_aromatic_), 7.20 (d, C═CH, J = 15.6 Hz), 7.08 (d, C═CH, J=15.0 Hz), 6.51 (s, 1H, C3, CH_thiophene_), 3.94 (s, OCH_3_), 3.73 (s, OCH_3_), 3.62 (s, OCH_3_),2.44 (s, 3H, –CH_3_), 2.17 (s, 3H, CH_3_).

### Refinement

The H-atoms were positioned geometrically (C–H = 0.93–0.96 Å) and refined as riding with *U*_iso_(H) = x*U*_eq_(C), where x = 1.5 for methyl and x = 1.2 for aryl H-atoms.

### Crystal data

**Table:**

C_18_H_20_O_4_S	*D*_x_ = 1.287 Mg m^−^^3^
*M**_r_* = 332.40	Mo *K*α radiation, λ = 0.71073 Å
Tetragonal, *I*4_1_/*a*	Cell parameters from 2225 reflections
Hall symbol: -I 4ad	θ = 2.1–25.2°
*a* = 19.5263 (5) Å	µ = 0.21 mm^−^^1^
*c* = 17.9952 (4) Å	*T* = 296 K
*V* = 6861.2 (3) Å^3^	Prism, yellow
*Z* = 16	0.26 × 0.18 × 0.16 mm
*F*(000) = 2816	

### Data collection

**Table:**

Bruker KAPPA APEXII CCD diffractometer	3106 independent reflections
Radiation source: fine-focus sealed tube	2225 reflections with *I* > 2σ(*I*)
graphite	*R*_int_ = 0.038
Detector resolution: 8.10 pixels mm^-1^	θ_max_ = 25.2°, θ_min_ = 2.1°
ω scans	*h* = −20→23
Absorption correction: multi-scan (*SADABS*; Bruker, 2005)	*k* = −23→23
*T*_min_ = 0.966, *T*_max_ = 0.975	*l* = −21→21
25995 measured reflections	

### Refinement

**Table:**

Refinement on *F*^2^	Primary atom site location: structure-invariant direct methods
Least-squares matrix: full	Secondary atom site location: difference Fourier map
*R*[*F*^2^ > 2σ(*F*^2^)] = 0.040	Hydrogen site location: inferred from neighbouring sites
*wR*(*F*^2^) = 0.113	H-atom parameters constrained
*S* = 1.05	*w* = 1/[σ^2^(*F*_o_^2^) + (0.0466*P*)^2^ + 4.0141*P*] where *P* = (*F*_o_^2^ + 2*F*_c_^2^)/3
3106 reflections	(Δ/σ)_max_ = 0.001
213 parameters	Δρ_max_ = 0.19 e Å^−^^3^
0 restraints	Δρ_min_ = −0.15 e Å^−^^3^

### Special details

**Table:**

Geometry. Bond distances, angles *etc*. have been calculated using the rounded fractional coordinates. All su's are estimated from the variances of the (full) variance-covariance matrix. The cell e.s.d.'s are taken into account in the estimation of distances, angles and torsion angles
Refinement. Refinement of *F*^2^ against ALL reflections. The weighted *R*-factor *wR* and goodness of fit *S* are based on *F*^2^, conventional *R*-factors *R* are based on *F*, with *F* set to zero for negative *F*^2^. The threshold expression of *F*^2^ > σ(*F*^2^) is used only for calculating *R*-factors(gt) *etc*. and is not relevant to the choice of reflections for refinement. *R*-factors based on *F*^2^ are statistically about twice as large as those based on *F*, and *R*- factors based on ALL data will be even larger.

### Fractional atomic coordinates and isotropic or equivalent isotropic displacement parameters (Å^2^)

**Table:**

	*x*	*y*	*z*	*U*_iso_*/*U*_eq_	
S1	0.35094 (3)	0.20431 (3)	0.27680 (3)	0.0714 (2)	
O1	0.12845 (9)	0.01126 (9)	0.64860 (7)	0.0807 (6)	
O2	−0.03254 (9)	−0.15317 (9)	0.54859 (9)	0.0813 (6)	
O3	0.00824 (9)	−0.12701 (10)	0.41648 (8)	0.0862 (7)	
O4	0.27754 (9)	0.14060 (9)	0.50556 (8)	0.0818 (6)	
C1	0.12078 (10)	−0.00807 (10)	0.52048 (10)	0.0514 (7)	
C2	0.09748 (11)	−0.02371 (11)	0.59198 (10)	0.0556 (7)	
C3	0.04661 (11)	−0.07194 (11)	0.60327 (11)	0.0600 (7)	
C4	0.01787 (11)	−0.10560 (11)	0.54363 (12)	0.0600 (8)	
C5	0.04047 (11)	−0.09097 (12)	0.47106 (11)	0.0604 (8)	
C6	0.09028 (11)	−0.04341 (11)	0.46095 (11)	0.0569 (7)	
C7	0.10721 (14)	−0.00049 (14)	0.72270 (11)	0.0754 (9)	
C8	−0.05983 (14)	−0.16834 (16)	0.61946 (14)	0.0904 (11)	
C9	0.03306 (14)	−0.12099 (15)	0.34337 (12)	0.0829 (10)	
C10	0.17351 (10)	0.04257 (11)	0.51010 (10)	0.0553 (7)	
C11	0.20201 (11)	0.06423 (11)	0.44711 (11)	0.0581 (7)	
C12	0.25438 (11)	0.11732 (11)	0.44709 (11)	0.0579 (7)	
C13	0.28009 (11)	0.14298 (11)	0.37456 (10)	0.0536 (7)	
C14	0.25098 (12)	0.12648 (12)	0.30381 (11)	0.0622 (8)	
C15	0.28342 (12)	0.15590 (12)	0.24581 (11)	0.0636 (8)	
C16	0.26802 (14)	0.14998 (15)	0.16401 (12)	0.0857 (10)	
C17	0.33529 (11)	0.18552 (11)	0.36833 (11)	0.0577 (7)	
C18	0.38074 (14)	0.21495 (14)	0.42741 (13)	0.0810 (10)	
H3	0.03178	−0.08165	0.65123	0.0720*	
H6	0.10480	−0.03382	0.41285	0.0683*	
H7A	0.05890	0.00783	0.72682	0.1132*	
H7B	0.13148	0.02983	0.75542	0.1132*	
H7C	0.11684	−0.04708	0.73613	0.1132*	
H8A	−0.02460	−0.18752	0.65031	0.1355*	
H8B	−0.09654	−0.20070	0.61437	0.1355*	
H8C	−0.07688	−0.12709	0.64183	0.1355*	
H9A	0.02961	−0.07418	0.32751	0.1242*	
H9B	0.00640	−0.14950	0.31093	0.1242*	
H9C	0.08010	−0.13520	0.34178	0.1242*	
H10	0.18992	0.06293	0.55329	0.0664*	
H11	0.18819	0.04507	0.40224	0.0697*	
H14	0.21311	0.09805	0.29830	0.0747*	
H16A	0.30512	0.12692	0.13955	0.1286*	
H16B	0.26257	0.19491	0.14319	0.1286*	
H16C	0.22655	0.12433	0.15719	0.1286*	
H18A	0.35531	0.24738	0.45650	0.1215*	
H18B	0.41902	0.23751	0.40458	0.1215*	
H18C	0.39698	0.17880	0.45898	0.1215*	

### Atomic displacement parameters (Å^2^)

**Table:**

	*U*^11^	*U*^22^	*U*^33^	*U*^12^	*U*^13^	*U*^23^
S1	0.0843 (4)	0.0798 (4)	0.0502 (3)	−0.0019 (3)	0.0108 (3)	0.0054 (3)
O1	0.1053 (13)	0.0996 (12)	0.0372 (7)	−0.0388 (10)	−0.0040 (8)	−0.0007 (8)
O2	0.0825 (11)	0.0952 (12)	0.0662 (10)	−0.0260 (10)	0.0093 (8)	−0.0140 (9)
O3	0.0853 (12)	0.1182 (14)	0.0552 (9)	−0.0282 (10)	0.0079 (8)	−0.0337 (9)
O4	0.0953 (12)	0.1074 (13)	0.0426 (8)	−0.0294 (10)	−0.0032 (8)	−0.0034 (8)
C1	0.0545 (12)	0.0567 (12)	0.0430 (10)	0.0055 (10)	−0.0006 (8)	−0.0037 (9)
C2	0.0648 (13)	0.0599 (12)	0.0421 (10)	−0.0002 (11)	−0.0039 (9)	−0.0020 (9)
C3	0.0685 (14)	0.0697 (14)	0.0417 (10)	−0.0026 (12)	0.0018 (9)	0.0004 (10)
C4	0.0582 (13)	0.0642 (14)	0.0575 (12)	−0.0024 (11)	0.0040 (10)	−0.0072 (10)
C5	0.0594 (13)	0.0720 (14)	0.0497 (12)	0.0016 (12)	0.0015 (10)	−0.0171 (10)
C6	0.0582 (12)	0.0691 (14)	0.0434 (10)	0.0050 (11)	0.0060 (9)	−0.0084 (9)
C7	0.0982 (18)	0.0905 (17)	0.0376 (11)	−0.0146 (14)	−0.0026 (11)	0.0011 (10)
C8	0.093 (2)	0.104 (2)	0.0743 (16)	−0.0267 (17)	0.0136 (14)	0.0047 (14)
C9	0.0979 (19)	0.100 (2)	0.0507 (13)	−0.0002 (16)	−0.0021 (12)	−0.0240 (12)
C10	0.0624 (13)	0.0620 (13)	0.0416 (10)	0.0028 (10)	−0.0034 (9)	−0.0044 (9)
C11	0.0659 (13)	0.0673 (14)	0.0411 (10)	−0.0020 (11)	−0.0016 (9)	−0.0048 (9)
C12	0.0644 (13)	0.0685 (14)	0.0408 (10)	0.0026 (11)	−0.0012 (9)	−0.0016 (9)
C13	0.0584 (12)	0.0586 (12)	0.0439 (10)	0.0069 (10)	0.0009 (9)	0.0003 (9)
C14	0.0656 (14)	0.0786 (15)	0.0425 (11)	0.0027 (11)	−0.0005 (10)	−0.0003 (10)
C15	0.0700 (14)	0.0767 (15)	0.0440 (11)	0.0121 (12)	0.0002 (10)	−0.0011 (10)
C16	0.0957 (19)	0.120 (2)	0.0414 (12)	0.0123 (16)	−0.0012 (12)	0.0040 (12)
C17	0.0666 (14)	0.0611 (13)	0.0454 (11)	0.0065 (11)	0.0041 (9)	−0.0021 (9)
C18	0.0866 (18)	0.0967 (19)	0.0597 (14)	−0.0225 (15)	0.0038 (12)	−0.0086 (13)

### Geometric parameters (Å, °)

**Table:**

S1—C15	1.715 (2)	C15—C16	1.507 (3)
S1—C17	1.715 (2)	C17—C18	1.499 (3)
O1—C2	1.368 (2)	C3—H3	0.9300
O1—C7	1.415 (2)	C6—H6	0.9300
O2—C4	1.356 (3)	C7—H7A	0.9600
O2—C8	1.414 (3)	C7—H7B	0.9600
O3—C5	1.362 (3)	C7—H7C	0.9600
O3—C9	1.407 (3)	C8—H8A	0.9600
O4—C12	1.232 (3)	C8—H8B	0.9600
C1—C2	1.399 (3)	C8—H8C	0.9600
C1—C6	1.407 (3)	C9—H9A	0.9600
C1—C10	1.440 (3)	C9—H9B	0.9600
C2—C3	1.384 (3)	C9—H9C	0.9600
C3—C4	1.378 (3)	C10—H10	0.9300
C4—C5	1.408 (3)	C11—H11	0.9300
C5—C6	1.357 (3)	C14—H14	0.9300
C10—C11	1.332 (3)	C16—H16A	0.9600
C11—C12	1.456 (3)	C16—H16B	0.9600
C12—C13	1.486 (3)	C16—H16C	0.9600
C13—C14	1.431 (3)	C18—H18A	0.9600
C13—C17	1.365 (3)	C18—H18B	0.9600
C14—C15	1.349 (3)	C18—H18C	0.9600
			
O2···O3	2.559 (2)	H6···C9	2.5300
O2···C9^i^	3.410 (3)	H6···C11	2.7700
O3···O2	2.559 (2)	H6···H9A	2.2700
O3···C9^i^	3.209 (3)	H6···H9C	2.4100
O4···C18	2.854 (3)	H6···H11	2.2500
O1···H10	2.3200	H6···H10^viii^	2.5900
O3···H9C^i^	2.5500	H7A···C3	2.7300
O4···H10	2.4400	H7A···H3	2.2800
O4···H18A	2.7300	H7A···H9A^iv^	2.3700
O4···H18C	2.5900	H7B···C16^v^	2.9300
O4···H8C^ii^	2.7600	H7B···H16A^v^	2.4700
O4···H9A^iii^	2.8400	H7B···C1^iii^	2.9100
O4···H14^iii^	2.5700	H7B···C6^iii^	3.0400
C4···C6^iv^	3.596 (3)	H7C···C3	2.8000
C6···C4^iv^	3.596 (3)	H7C···H3	2.3600
C7···C16^v^	3.464 (3)	H7C···C15^ix^	2.8900
C9···O3^vi^	3.209 (3)	H8A···C3	2.7800
C9···O2^vi^	3.410 (3)	H8A···H3	2.3400
C16···C7^vii^	3.464 (3)	H8C···C3	2.7300
C18···O4	2.854 (3)	H8C···H3	2.3100
C1···H7B^viii^	2.9100	H8C···O4^xiii^	2.7600
C2···H16A^ix^	2.9000	H9A···C6	2.7400
C3···H8C	2.7300	H9A···H6	2.2700
C3···H8A	2.7800	H9A···H7A^iv^	2.3700
C3···H7A	2.7300	H9A···O4^viii^	2.8400
C3···H7C	2.8000	H9C···C6	2.8000
C6···H7B^viii^	3.0400	H9C···H6	2.4100
C6···H9C	2.8000	H9C···O3^vi^	2.5500
C6···H11	2.7800	H10···O1	2.3200
C6···H9A	2.7400	H10···O4	2.4400
C7···H3	2.5200	H10···H6^iii^	2.5900
C8···H3	2.5300	H11···C6	2.7800
C9···H6	2.5300	H11···C14	2.6800
C11···H6	2.7700	H11···H6	2.2500
C11···H14	2.7700	H11···H14	2.1900
C12···H14^iii^	3.0200	H14···C11	2.7700
C12···H18C	3.0400	H14···H11	2.1900
C12···H16C^iii^	3.0600	H14···O4^viii^	2.5700
C13···H16C^iii^	3.0300	H14···C12^viii^	3.0200
C14···H11	2.6800	H16A···C2^x^	2.9000
C15···H7C^x^	2.8900	H16A···H7B^vii^	2.4700
C16···H7B^vii^	2.9300	H16B···C17^xi^	3.0200
C17···H16B^xi^	3.0200	H16C···C12^viii^	3.0600
C18···H18B^xii^	3.1000	H16C···C13^viii^	3.0300
H3···C7	2.5200	H18A···O4	2.7300
H3···C8	2.5300	H18B···C18^xiv^	3.1000
H3···H7A	2.2800	H18B···H18B^xiv^	2.5000
H3···H7C	2.3600	H18B···H18B^xii^	2.5000
H3···H8A	2.3400	H18C···O4	2.5900
H3···H8C	2.3100	H18C···C12	3.0400
			
C15—S1—C17	93.29 (10)	O1—C7—H7A	109.00
C2—O1—C7	119.44 (18)	O1—C7—H7B	109.00
C4—O2—C8	118.45 (19)	O1—C7—H7C	109.00
C5—O3—C9	118.16 (19)	H7A—C7—H7B	109.00
C2—C1—C6	117.12 (18)	H7A—C7—H7C	109.00
C2—C1—C10	120.13 (17)	H7B—C7—H7C	109.00
C6—C1—C10	122.75 (17)	O2—C8—H8A	109.00
O1—C2—C1	115.63 (18)	O2—C8—H8B	109.00
O1—C2—C3	123.22 (17)	O2—C8—H8C	109.00
C1—C2—C3	121.15 (18)	H8A—C8—H8B	109.00
C2—C3—C4	120.17 (19)	H8A—C8—H8C	109.00
O2—C4—C3	124.82 (19)	H8B—C8—H8C	109.00
O2—C4—C5	115.31 (19)	O3—C9—H9A	109.00
C3—C4—C5	119.9 (2)	O3—C9—H9B	109.00
O3—C5—C4	114.78 (19)	O3—C9—H9C	109.00
O3—C5—C6	126.01 (19)	H9A—C9—H9B	109.00
C4—C5—C6	119.20 (19)	H9A—C9—H9C	109.00
C1—C6—C5	122.49 (18)	H9B—C9—H9C	109.00
C1—C10—C11	128.86 (18)	C1—C10—H10	116.00
C10—C11—C12	121.32 (19)	C11—C10—H10	116.00
O4—C12—C11	121.34 (19)	C10—C11—H11	119.00
O4—C12—C13	120.12 (19)	C12—C11—H11	119.00
C11—C12—C13	118.53 (18)	C13—C14—H14	123.00
C12—C13—C14	124.86 (19)	C15—C14—H14	123.00
C12—C13—C17	122.97 (18)	C15—C16—H16A	109.00
C14—C13—C17	112.18 (18)	C15—C16—H16B	109.00
C13—C14—C15	114.0 (2)	C15—C16—H16C	109.00
S1—C15—C14	110.12 (16)	H16A—C16—H16B	109.00
S1—C15—C16	120.88 (17)	H16A—C16—H16C	109.00
C14—C15—C16	129.0 (2)	H16B—C16—H16C	109.00
S1—C17—C13	110.47 (15)	C17—C18—H18A	109.00
S1—C17—C18	119.58 (17)	C17—C18—H18B	109.00
C13—C17—C18	129.96 (19)	C17—C18—H18C	109.00
C2—C3—H3	120.00	H18A—C18—H18B	109.00
C4—C3—H3	120.00	H18A—C18—H18C	109.00
C1—C6—H6	119.00	H18B—C18—H18C	109.00
C5—C6—H6	119.00		
			
C17—S1—C15—C14	−0.40 (19)	O2—C4—C5—O3	−0.1 (3)
C17—S1—C15—C16	−179.6 (2)	O2—C4—C5—C6	179.3 (2)
C15—S1—C17—C13	0.38 (18)	C3—C4—C5—O3	−179.7 (2)
C15—S1—C17—C18	179.9 (2)	C3—C4—C5—C6	−0.3 (3)
C7—O1—C2—C1	178.9 (2)	O3—C5—C6—C1	179.6 (2)
C7—O1—C2—C3	−1.5 (3)	C4—C5—C6—C1	0.3 (3)
C8—O2—C4—C3	2.2 (3)	C1—C10—C11—C12	178.9 (2)
C8—O2—C4—C5	−177.3 (2)	C10—C11—C12—O4	5.2 (3)
C9—O3—C5—C4	−173.0 (2)	C10—C11—C12—C13	−175.2 (2)
C9—O3—C5—C6	7.6 (3)	O4—C12—C13—C14	−171.4 (2)
C6—C1—C2—O1	179.65 (19)	O4—C12—C13—C17	8.8 (3)
C6—C1—C2—C3	0.0 (3)	C11—C12—C13—C14	9.0 (3)
C10—C1—C2—O1	−0.7 (3)	C11—C12—C13—C17	−170.8 (2)
C10—C1—C2—C3	179.7 (2)	C12—C13—C14—C15	−179.9 (2)
C2—C1—C6—C5	−0.2 (3)	C17—C13—C14—C15	0.0 (3)
C10—C1—C6—C5	−179.8 (2)	C12—C13—C17—S1	179.55 (17)
C2—C1—C10—C11	−180.0 (2)	C12—C13—C17—C18	0.2 (4)
C6—C1—C10—C11	−0.3 (3)	C14—C13—C17—S1	−0.3 (2)
O1—C2—C3—C4	−179.6 (2)	C14—C13—C17—C18	−179.7 (2)
C1—C2—C3—C4	0.0 (3)	C13—C14—C15—S1	0.3 (3)
C2—C3—C4—O2	−179.4 (2)	C13—C14—C15—C16	179.4 (2)
C2—C3—C4—C5	0.2 (3)		

Symmetry codes: (i) −*y*−1/4, *x*−1/4, −*z*+3/4; (ii) −*y*+1/4, *x*+1/4, −*z*+5/4; (iii) *y*+1/4, −*x*+1/4, *z*+1/4; (iv) −*x*, −*y*, −*z*+1; (v) −*y*+1/4, *x*−1/4, *z*+3/4; (vi) *y*+1/4, −*x*−1/4, −*z*+3/4; (vii) *y*+1/4, −*x*+1/4, *z*−3/4; (viii) −*y*+1/4, *x*−1/4, *z*−1/4; (ix) −*x*+1/2, −*y*, *z*+1/2; (x) −*x*+1/2, −*y*, *z*−1/2; (xi) −*x*+1/2, −*y*+1/2, −*z*+1/2; (xii) −*y*+3/4, *x*−1/4, −*z*+3/4; (xiii) *y*−1/4, −*x*+1/4, −*z*+5/4; (xiv) *y*+1/4, −*x*+3/4, −*z*+3/4.

### Hydrogen-bond geometry (Å, °)

**Table:**

*D*—H···*A*	*D*—H	H···*A*	*D*···*A*	*D*—H···*A*
C9—H9C···O3^vi^	0.96	2.55	3.209 (3)	126
C14—H14···O4^viii^	0.93	2.57	3.483 (3)	168

Symmetry codes: (vi) *y*+1/4, −*x*−1/4, −*z*+3/4; (viii) −*y*+1/4, *x*−1/4, *z*−1/4.
